# Identification of MicroRNAs and Their Targets That Respond to Powdery Mildew Infection in Cucumber by Small RNA and Degradome Sequencing

**DOI:** 10.3389/fgene.2020.00246

**Published:** 2020-03-26

**Authors:** Xuewen Xu, Cailian Zhong, Min Tan, Ya Song, Xiaohua Qi, Qiang Xu, Xuehao Chen

**Affiliations:** ^1^School of Horticulture and Plant Protection, Yangzhou University, Yangzhou, China; ^2^Joint International Research Laboratory of Agriculture & Agri-Product Safety, Yangzhou University, Yangzhou, China; ^3^State Key Laboratory of Vegetable Germplasm Innovation, Tianjin, China

**Keywords:** cucumber, powdery mildew, miRNA, target genes, comparative analysis

## Abstract

Powdery mildew (PM) is a prevalent disease known to limit cucumber production worldwide. MicroRNAs (miRNAs) are single-stranded molecules that regulate host defense responses through posttranscriptional gene regulation. However, which specific miRNAs are involved and how they regulate cucumber PM resistance remain elusive. A PM-resistant single-segment substitution line, SSSL508-28, was developed previously using marker-assisted backcrossing of the PM-susceptible cucumber inbred D8 line. In this study, we applied small RNA and degradome sequencing to identify PM-responsive miRNAs and their target genes in the D8 and SSSL508-28 lines. The deep sequencing resulted in the identification of 156 known and 147 novel miRNAs. Among them, 32 and six differentially expressed miRNAs (DEMs) were detected in D8 and SSSL508-28, respectively. The positive correlation between DEMs measured by small RNA sequencing and stem-loop quantitative real-time reverse transcription–polymerase chain reaction confirmed the accuracy of the observed miRNA abundances. The 32 DEMs identified in the PM-susceptible D8 were all upregulated, whereas four of the six DEMs identified in the PM-resistant SSSL508-28 were downregulated. Using *in silico* and degradome sequencing approaches, 517 and 20 target genes were predicted for the D8 and SSSL508-28 DEMs, respectively. Comparison of the DEM expression profiles with the corresponding mRNA expression profiles obtained in a previous study with the same experimental design identified 60 and three target genes in D8 and SSSL508-28, respectively, which exhibited inverse expression patterns with their respective miRNAs. In particular, five DEMs were located in the substituted segment that contained two upregulated DEMs, Csa-miR172c-3p and Csa-miR395a-3p, in D8 and two downregulated DEMs, Csa-miR395d-3p and Csa-miR398b-3p, in SSSL508-28. One gene encoding L-aspartate oxidase, which was targeted by Csa-miR162a, was also located on the same segment and was specifically downregulated in PM-inoculated D8 leaves. Our results will facilitate the future use of miRNAs in breeding cucumber varieties with enhanced resistance to PM.

## Introduction

Powdery mildew (PM) fungi are obligate biotrophic parasites that are spread worldwide and cause severe diseases in a large variety of horticultural plants, such as cucurbits, tomato, pea, and okra (Martínez-Cruz et al., 2017; Cobos et al., 2018; Fan et al., 2019). *Podosphaera xanthii* is the main cause of PM on cucumber under glasshouses and in open fields, which causes serious losses in crop yield and quality (Xu et al., 2016). The disease initiates as thin white spots, first on the surfaces of older leaves and then gradually spreading to younger leaves. Grayish white mycelia are visible on severely affected leaves, and such leaves finally exhibit chlorosis and senescence. The application of fungicide can effectively control the disease at present but is not environmentally friendly and is harmful to consumers’ health because cucumbers are harvested almost daily for fresh market consumption (Fukino et al., 2013; Xu et al., 2016). Genetic strategies to breed PM-resistant cultivars are currently the most promising and economical ways of controlling this disease. Thus, a deeper understanding of the molecular mechanisms that trigger cucumber plants to recognize and eventually prevent or limit PM infection is required.

Genetic mapping, RNA sequencing-based transcriptomic, and iTRAQ-based proteomic studies have revealed candidate genes and pathways associated with the genetic control and molecular responses of host immunity against cucumber PM. They include genes that encode cysteine-rich receptor-like protein kinases (*Csa1G064780* and *Csa1G064790*) and mildew locus O (*Csa5M623470*), as well as molecular events involving cell wall modifications, salicylic acid metabolism, and signal transduction pathways (Berg et al., 2015; Xu et al., 2016; Xu et al., 2017). However, little is known about the coordination and regulation of host genes that affect these biological processes. Studies of microRNAs (miRNAs) in plant species have found strong evidence that some miRNAs that are responsive to pathogen infections may be critical in regulating the host defenses (Islam et al., 2017). However, more investigations are needed to understand which and how these miRNAs contribute to the dialogue between plant and pathogen attack (Carrera et al., 2009; Sanz-Carbonell et al., 2019). MiRNAs are a group of 18 to 24 long nucleotides (nt) that are endogenous small single-stranded non-coding RNAs that regulate gene expression by repressing protein translation or by targeting mRNAs for degradation (Lin et al., 2016). Genome-wide analysis of miRNAs showed that eight wheat miRNAs from six families (miR394, miR528, miR396, miR171, miR156, and miR160) were differentially expressed upon PM (*Blumeria graminis* f. sp. *tritici Bgt*) infection (Wu et al., 2015). More recently, miRNAs related to the response of barley to PM (*B. graminis* f. sp. *hordei*) were putatively identified as regulating the transcript levels of transcription factors (TFs), such as auxin response factors, NAC [for NAM (no apical meristem), ATAF, CUC (cup-shaped cotyledon)], and homeodomains, as well as several splicing factors (Hunt et al., 2019).

Because miRNAs have been shown to be involved in PM infection, we hypothesized that miRNAs may also play a role during *P. xanthii* parasitism of its cucumber host and that they may exhibit different expression patterns between resistant and susceptible lines. To test the hypothesis, we inoculated cucumber seedlings from the D8 and SSSL508-28 lines with *P. xanthii*. D8 is PM susceptible, and SSSL508-28, which has the genetic background of D8 but contains a single segment from PM-resistant line Jin5-508, is PM resistant. The extracted RNA from cucumber leaves 2 ‘s after inoculation was used for small RNA (sRNA) and degradome sequencing to capture cucumber PM-responsive miRNAs and their corresponding target genes. The results will widen our understanding of PM-responsive miRNA-mediated regulatory mechanisms in Cucurbitaceae plants.

## Materials and Methods

### Plant Material and Treatment

Cucumber seedlings of D8 and SSSL508-28 were grown in a growth chamber under controlled conditions (65% relative humidity, 28°C/16 h light and 20°C/8 h dark). At 21 days (three-leaf stage), homogeneous plants were selected for inoculation by spraying with a *P. xanthii* conidia (harvested from naturally infected D8 leaves) spore suspension of 1 × 10^6^ conidia/mL containing 0.01% Tween-20 or sterile distilled water until runoff (Xu et al., 2017). The leaves sprayed with the sterile water served as mock controls. An average relative humidity of 90–100% was maintained after inoculation. The PM-inoculated and control leaves with three biological replicates were harvested 2 days after treatment, quickly frozen in liquid nitrogen, and maintained at −80°C until used for RNA isolation.

### Small RNA Dequencing and Data Processing

Total RNA was extracted from 10 pooled cucumber leaves using Trizol reagent (Invitrogen, Carlsbad, CA, United States). RNA quality was assessed using an Agilent Bioanalyzer 2100 (Agilent Technologies, Santa Clara, CA, United States), and samples with RNA integrity ≥8 were used to generate the sRNA libraries. Twelve sRNA libraries were generated following the protocol of the TruSeq sRNA Sample Prep Kits (Illumina, San Diego, CA, United States). Briefly, the RNA fragments that were 18 to 30 nt were enriched by polyacrylamide gel electrophoresis and ligated to 5′ and 3′ adaptors using T4 RNA ligase. The ligated RNAs were reverse transcribed to generate the cDNAs libraries. Single-end sequencing (50 bp) was performed on an Illumina HiSeq 2500 platform (Illumina) by Biomarker Technologies (Beijing, China).

Clean reads were obtained from the raw reads by removing low-quality and adaptor-containing reads using the FASTAX-Toolkit. Clean reads that were 18- to 30-nt long were retained and aligned to the cucumber draft genome assembly (9930 V2.0^1^), Rfam 14.1,^2^ Silva,^3^ GtRNAdb,^4^ and Repbase^5^ to identify non-coding RNAs using Bowtie (Langmead et al., 2009). The remaining clean reads were mapped to the miRBase database (release 22^6^) to identify known cucumber miRNAs. Up to two mismatches were allowed in the alignments. The remaining unaligned sequences were analyzed using MTide, a probabilistic model-based miRNA prediction software especially designed for plant miRNAs (Zhang et al., 2014), to predict novel miRNAs. The cucumber genome (9930 V2.0) was used as the reference genome. The novel miRNAs that met the following stringent criteria were retained: (1) sequence length 18 to 25 nt; (2) maximal free energy of the miRNA precursor −20 kcal/mol; (3) presence of star sequences and 3′ overhang; and (4) ≤7 nucleotide mismatches between the miRNA and star miRNA. The potential novel miRNA precursors that aligned with tRNA, rRNA, snRNA, or snoRNA sequences were discarded.

The abundance of identified miRNAs in the different samples was normalized to transcripts per kilobase million (TPM). The R-based DESeq2 package (Love et al., 2014) was used to determine differential expression patterns of the identified miRNAs between PM-inoculated and control leaves. An miRNA was considered to be significantly differentially expressed if it exhibited a fold change ≥1.5 or ≤0.67 with *p* ≤ 0.05.

### Degradome Sequencing and Data Processing

Four libraries were constructed for degradome sequencing with the RNA extracted from PM-inoculated D8, PM-inoculated SSSL508-28, and the control D8 and SSSL508-28 leaves. An Oligotex mRNA mini kit (Qiagen, Hilden, Germany) was used to separate poly(A) +RNA from the total RNA. The poly(A) +RNA was bound to the mRNACapture beads. A 5′-adaptor was ligated to the RNAs with 5′ monophosphates, followed by reverse transcription with 3′-random primers and polymerase chain reaction (PCR) amplification. The gel-purified products were sequenced using only the 5′-adaptor ligated sequences. Single-end sequencing (36 bp) of the degradome libraries also was performed on an Illumina HiSeq 2500 platform by Beijing Biomarker Technologies (Beijing, China).

After trimming, the clean reads that matched sequences in GenBank and Rfam 14.1 were removed. The remaining 20- or 21-nt high-quality reads were aligned to the cucumber cDNA sequences (9930 V2.0). Perfectly matched sequences were processed using the CleaveLand pipeline (v4.4) to predict potential cleavage sites (Addo-Quaye et al., 2008).

### Identification and Annotation of Target Genes

The psRNATarget server^7^ was used to predict the miRNA-mediated target genes. We used the cucumber miRNA sequences as query sequences. The cucumber draft genome assembly (cDNA, 9930 V2.0) was used as the reference database. BLASTn hits with fewer than three mismatches were selected as potential targets. Gene ontology (GO) slim classification analysis was performed using the gene classification toolkit in CuGenDB.^8^

### Stem-Loop Quantitative Real-Time Reverse Transcription–PCR Analysis

To validate the differential expressions of miRNA identified by sRNA sequencing (sRNA-seq), quantification of selected miRNAs was performed by stem-loop quantitative real-time reverse transcription (qRT)–PCR analysis. Synthesis of first-strand cDNA and the qRT-PCRs were performed using the miRcute fluorescent quantitative detection (SYBR Green) kit (FP401; Tiangen, Beijing, China). Primers were designed using the Vazyme miRNA designer (V1.01; Nanjing, China). The primer sequences are listed in Table 1. The PCRs were performed on an iQ5 multicolor real-time PCR detection instrument (Bio-Rad, Hercules, CA, United States). The relative expressions of the miRNAs were calculated using the 2^–ΔΔ*CT*^ method with U6 snRNA as the internal control. The PCRs were performed in triplicate. A correlation analysis between the expressions obtained by qRT-PCR and the expression levels obtained using the sRNA-seq data was performed using SAS 9.0 software (SAS Institute, Inc., Cary, NC, United States).

**TABLE 1:** Primers used for the stem-loop qPCRs in this study.

#	miRNAs	Primer sequence (5′-3′)
1	Csa-miR156g	F:GCGCGGACAGAAGAGAGG
2	Csa-miR171g	F:CGCGCGGAGAGCCGCG
3	Csa-miR160b	F:GCGCGCGGCCGGCCCC
4	Csa-miR162a	F:GCGCGCGCGAAAACCC
5	Csa-miR396d-3p	F:GCGCGCGCAAAAAGCG
6	Csa-miR395d-3p	F:GCGCGCGCGAAGGGGG
7	Csa-novel_71	F:GGCGGTTCGGTTTGGTTTA
8	Stem sequence	GTCGTATCCAGTGCAGGGTCCGAGGTAT TCGCACTGGATACGAC
9	U6	F:GGGGACATCCGATAAAATT

## Results

### Symptoms of PM Infection on SSSL508-28 and D8 Leaves

**FIGURE 1:**
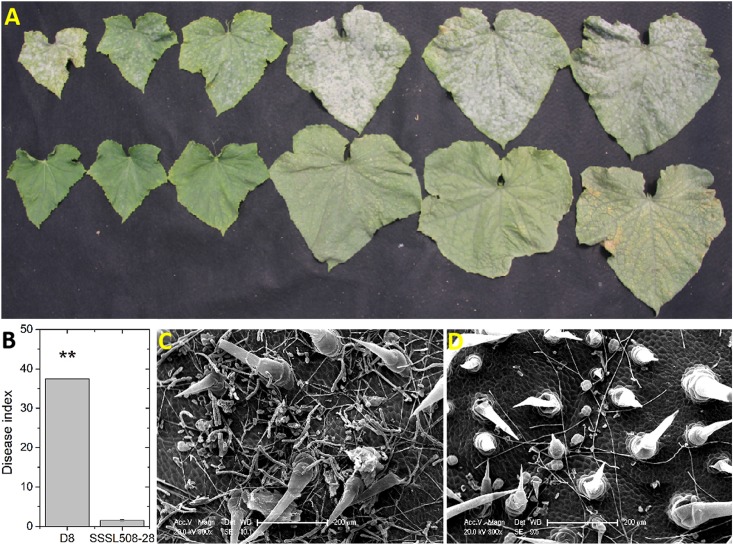
Performance of cucumber SSSL508-28 and D8 lines infected with PM. **(A)** Typical symptoms on cucumber leaves in different growth stages upon infection with the PM pathogen. Upper: D8; Lower: SSSL508-28. **(B)** Disease indexes of the two lines upon infection with the PM pathogen. Phenotypes of D8 **(C)** and SSSL508-28 **(D)** leaves 48 h after PM inoculation by scanning electron microscopy.

Representative symptoms to PM infection on D8 and SSSL508-28 leaves are shown in Figure 1A. The mean disease indexes of D8 and SSSL508-28 were 37.3 and 2.1, respectively (Figure 1B). The phenotypic differences between the PM-resistant and PM-susceptible lines were determined by observing the extent of PM growth on the leaf surface at 2 days post PM inoculation by scanning electron microscopy. Dense PM hyphae were seen on the surface of the D8 leaves, whereas no conidia were detected on the surface of the SSSL508-28 leaves (Figure 1C). This suggested that differences in the resistance mechanisms between SSSL508-28 and D8 may contribute to the observed difference in phenotype.

### Sequencing the sRNA Libraries of Cucumber Leaves

**FIGURE 2:**
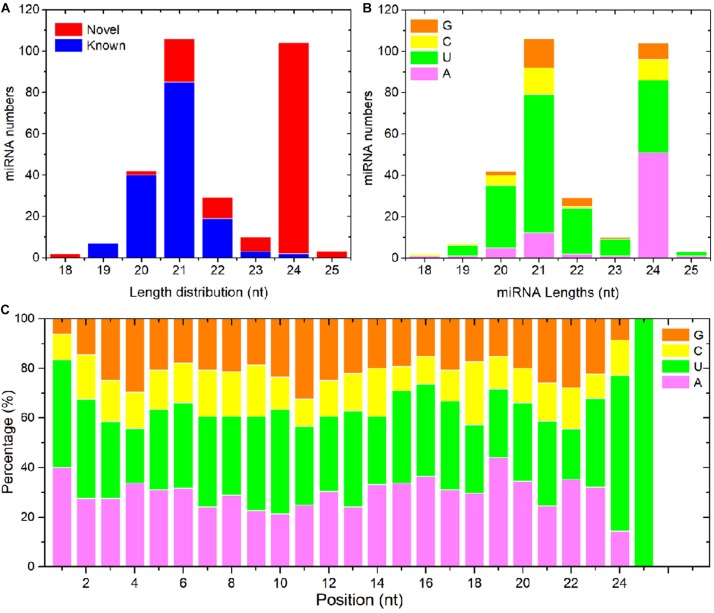
Length distribution and nucleotide bias of the detected cucumber miRNAs. **(A)** Length distribution of known and novel cucumber miRNAs. **(B)** Nucleotide composition of the mature miRNAs. **(C)** Nucleotide bias at each position of the mature miRNA sequences.

To detect miRNAs related to PM resistance, 12 sRNA libraries with three biological replicates were constructed for sRNA-seq. They contained the RNAs extracted from non-inoculated D8 control leaves, PM-inoculated D8 leaves, non-inoculated SSSL508-28 control leaves, and PM-inoculated SSSL508-28 leaves. The numbers of raw reads from each library were between 17.9 and 35.1 million (Table 2). After filtering low-quality reads, adaptor sequences, and reads <16 or >30 nt long, more than 13.5 million clean reads remained in each library (Table 2). After the non-coding RNAs (e.g. tRNAs, rRNAs, snRNAs, and snoRNAs) were removed, the remaining >2.49 million reads in each library had at least one match to the cucumber reference genome (Table 2). The length distribution of all the miRNAs that mapped to the reference genome is presented in Figure 2A. The 24-nt miRNAs were the most abundant in all 12 libraries, which is similar to previous findings in plant species such as *Arabidopsis*, rice, and soybean. Analysis of nucleotide bias in the 18- to 30-nt miRNAs showed that the 19- to 22-nt mature miRNAs preferentially began with uracil (U) (Figure 2B). In addition, analysis of specific nucleotide occurrence showed a dominant bias for U at the first two positions, and the frequency of cytosine (C) was always lowest at positions 2 to 24 in the mature miRNA sequences (Figure 2C).

**TABLE 2:** Statistics of the sRNA sequencing reads in the 12 libraries of cucumber leaves.

Sample	Total reads	Clean reads	Q30	Mapped sRNA
NID_1	29,896,281	24,643,689	98.68	4,542,524
NID_2	23,092,787	18,855,201	98.94	2,903,203
NID_3	24,086,823	20,411,802	98.9	5,381,892
ID_1	17,888,875	13,462,090	98.74	2,837,324
ID_2	30,359,661	19,939,452	98.89	3,743,139
ID_3	35,108,162	27,678,646	98.63	4,662,024
NIS_1	22,008,499	18,213,173	98.84	3,191,520
NIS_2	21,027,618	16,702,521	98.68	2,829,001
NIS_3	23,521,845	19,466,300	98.89	3,919,314
IS_1	26,547,109	21,369,809	99.09	2,610,609
IS_2	25,500,306	18,085,569	99.18	3,608,728
IS_3	23,297,499	17,451,893	99.08	2,494,420

*NID, non-inoculated D8 control; ID, powdery mildew (PM)-inoculated D8; NIS, non-inoculated SSSL508-28 control; IS, PM-inoculated SSSL508-28.*

### Identification of Known Cucumber miRNAs

To identify the known miRNAs, we aligned the 18- to 30-nt sRNA reads to the known plant miRNA sequences in miRBase release 22 using BLASTn. A total of 170 known miRNAs corresponding to 35 miRNA families, including miR156, miR160, miR171, miR396, and miR408, were identified from the 12 libraries (Supplementary Table 1). The miR156 family was the largest with 20 members, followed by miR166 with 16 members and miR171 with 13 members. The precursor miRNA sequences varied in length from 54 to 403 nt. Of the known miRNAs, 152 and 148 were common between the control and PM-inoculated D8 and SSSL508-28 leaves, respectively, suggesting that expression of the known miRNAs was stable in the two cucumber lines. We also found that members of the same miRNA family exhibited variations in expression levels. For example, Csa-miR156j and Csa-miR156h both belong to miR156 family, and TPM values of Csa-miR156j were 3,553 to 7,519, whereas the TPM values of Csa-miR156h were only 0 to 9.8 in the 12 libraries. These differences in expression levels among different members of the same miRNA family suggest their roles in the PM response are different. Further, six known miRNAs, Csa-miR156o-3p, Csa-miR156q-3p, Csa-miR156v, Csa-miR169k, Csa-miR172b-5p, and Csa-miR477a, were detected in only one of the 12 samples.

### Identification of Novel Cucumber miRNAs

A total of 133 novel miRNAs were identified from the 12 libraries (Supplementary Table 2), and all had star sequences. The novel miRNAs were named as Csa_novel-number. The lengths of the novel miRNAs varied from 18 to 25 nt, with 24 nt being the predominant length (102/133). The minimum free energies of their pre-miRNA hairpin structures were −192.5 to −20.9 kcal⋅mol^–1^ with an average of −53.2 kcal⋅mol^–1^, which corresponds to those of other plant miRNA precursors (Zeng et al., 2018). Csa-novel_12 was the most abundant novel miRNA with a TPM value of >52,000 in all 12 libraries. Forty-nine of the novel miRNAs clustered with known miRNA families (Supplementary Table 2), suggesting they may be recently evolved members of these families.

### Differentially Expressed miRNAs in PM-Inoculated Cucumber Leaves

The read counts for the detected miRNA varied from 0 to 224,965, normalized to 0 to 368,491 TPM, among the 12 libraries (Supplementary Tables 1, 2). Using a fold change ≥1.5 and *p* ≤ 0.05 as the threshold, we identified 32 differentially expressed miRNAs (DEMs), including 27 known and five novel miRNAs, by comparing the PM-inoculated D8 (ID) with the non-inoculated D8 (NID) libraries (Table 3). Only six DEMs, including three known and three novel miRNAs, were identified by comparing the PM-inoculated SSSL508-28 (IS) with the non-inoculated SSSL508-28 (NIS) libraries (Table 4). Interestingly, all of the 32 DEMs identified in the ID versus NID comparison were upregulated, whereas four of the six DEMs identified in the IS versus NIS comparison were downregulated. This result provides an important basis for further studies of the roles of miRNAs in cucumber PM resistance and confirms that miRNAs may have important roles during pathogen infection.

**TABLE 3:** Differentially expressed miRNAs identified in the cucumber D8 line.

#	miRNA	IDmean	NID mean	Fold change	*P*-value	Up/down
1	Csa-miR160c-3p	99.710	32.883	3.032	9.7E-05	Up
2	Csa-miR160a-3p	57.096	25.061	2.278	0.005	Up
3	Csa-miR162	2, 603.735	1, 532.232	1.699	0.007	Up
4	Csa-miR162a	2, 603.735	1, 532.232	1.699	0.007	Up
5	Csa-miR319i	366.395	206.636	1.773	0.012	Up
6	Csa-miR396i-3p	463.470	234.666	1.975	0.014	Up
7	Csa-miR156j	6, 146.003	3, 918.313	1.569	0.016	Up
8	Csa-miR156k	6, 099.0471	3, 904.161	1.562	0.016	Up
9	Csa-miR160f	111.573	59.084	1.889	0.016	Up
10	Csa_novel-3	50.628	23.553	2.149	0.017	Up
11	Csa-miR171k-3p	198.174	98.795	2.006	0.017	Up
12	Csa-miR396b-3p	232.832	153.288	1.519	0.018	Up
13	Csa-miR172c-3p	4.782	2.220	2.154	0.019	Up
14	Csa-miR858b	2, 757.065	1, 345.456	2.049	0.019	Up
15	Csa-miR171f-3p	335.735	206.353	1.627	0.022	Up
16	Csa-miR395a-3p	27.188	14.028	1.938	0.023	Up
17	Csa-miR169g	799.649	441.886	1.810	0.025	Up
18	Csa-miR171a-3p	352.567	218.658	1.612	0.026	Up
19	Csa-miR171g	352.567	218.658	1.612	0.026	Up
20	Csa-miR858a	4, 803.275	1, 970.359	2.438	0.027	Up
21	Csa-miR396d-3p	2, 914.175	1, 714.185	1.700	0.028	Up
22	Csa-miR166a-5p	1, 188.020	671.633	1.769	0.029	Up
23	Csa-miR396a-3p	3, 017.994	1, 830.974	1.648	0.030	Up
24	Csa-miR171m	161.008	72.697	2.215	0.030	Up
25	Csa-miR164f-5p	152.143	83.242	1.828	0.030	Up
26	Csa-miR408e	5, 689.822	2, 759.366	2.062	0.031	Up
27	Csa_novel-19	2, 589.925	1, 096.975	2.361	0.032	Up
28	Csa-miR160a-5p	324.906	200.441	1.621	0.036	Up
29	Csa-miR160b	322.066	200.681	1.605	0.038	Up
30	Csa-miR160h	324.077	202.452	1.601	0.039	Up
31	Csa-miR156e	1, 615.159	528.463	3.056	0.039	Up
32	Csa-miR156g	75.300	15.707	4.794	0.041	Up

*ID mean, mean TPM value of three replications of powdery mildew (PM)–inoculated D8 leaves; NID mean, mean TPM value of three replications of non-inoculated D8 control leaves.*

**TABLE 4:** Differentially expressed miRNAs identified in the cucumber SSSL508-28 line.

#	miRNA	IS mean	NIS mean	Fold change	*P*	Up/down
1	Csa-miR395d-3p	1.027	14.390	0.071	0.017	Down
2	Csa_novel-71	140.625	68.462	2.054	0.017	Up
3	Csa_novel-15	860.313	490.768	1.753	0.018	Up
4	Csa-miR398b-3p	83.922	152.486	0.550	0.023	Down
5	Csa_novel-11	21.054	34.100	0.617	0.029	Down
6	Csa-miR398c	40, 250.733	61, 930.021	0.650	0.037	Down

*IS mean, mean TPM value of three replications of powdery mildew (PM)–inoculated SSSL508-28 leaves; NIS mean, mean TPM value of three replications of non-inoculated SSSL508-28 control leaves.*

### Verification of the Expression of Cucumber PM-Responsive miRNAs

**FIGURE 3:**
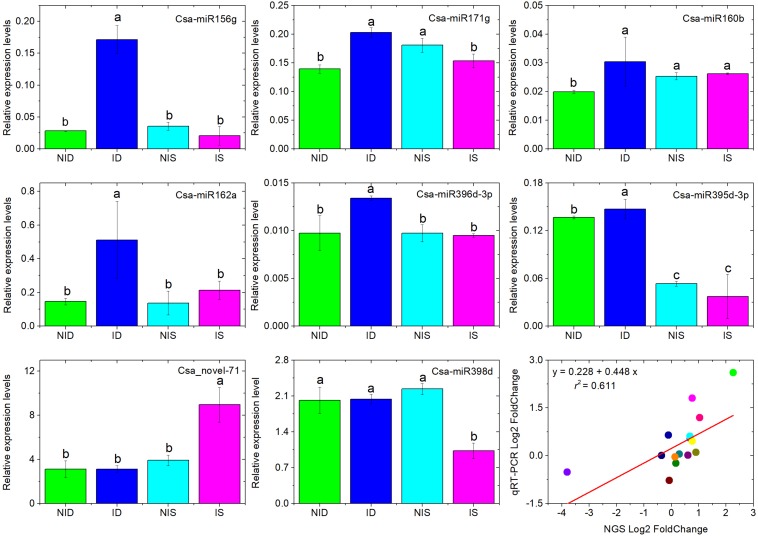
Analysis of eight selected miRNAs in cucumber SSSL508-28 and D8 lines 48 h after PM inoculation by stem-loop qRT-PCR. Data are the means of three replicates (±SD). The U6 gene was used as an internal control to normalize the expression data. Means with the same lowercase letter do not significantly differ by the least significant difference test at *p* ≤ 0.05 with a completely randomized design. Regression analysis between the eight miRNA expression levels measured by RNA sequencing and qRT-PCR indicates strong correlation between the two approaches with Pearson correlation coefficient *r*^2^ = 0.611. The gene expression values were transformed to the log2 scale.

To verify the identified DEMs obtained by sRNA-seq, seven known miRNAs (Csa-miR156g, Csa-miR171g, Csa-miR160b, Csa-miR162a, Csa-miR396d-3p, Csa-miR395d-3p, Csa-miR398d), and one novel miRNA (Csa-novel_71) were selected, and their expression levels were analyzed by stem-loop qRT-PCR. As shown in Figure 3, the stem-loop qRT-PCR showed that Csa-miR156g, Csa-miR171g, Csa-miR160b, Csa-miR162a, and Csa-miR396d-3p were upregulated only in PM-inoculated D8; Csa-miR398d was downregulated only in PM-inoculated SSSL508-28, and Csa-novel_71 was upregulated only in PM-inoculated SSSL508-28 compared with the corresponding non-inoculated controls. The Pearson correlation coefficient revealed a strong positive correlation (*r*^2^ = 0.611, *p* < 0.01) between the sRNA-seq and stem-loop qRT-PCR expression values, which confirmed the reliability of the sRNA-seq data. However, divergences between the two analyses were also observed.

For example, our stem-loop qRT-PCR experiment showed that Csa-miR171g was downregulated in PM-inoculated SSSL508-28, whereas no significant difference was found from the sequencing results.

The discrepancies were also previously described and may be ascribed to sequence bias introduced by sRNA libraries or profiling stem-loop qRT-PCR or to different normalization approaches employed in these two strategies (Pantaleo et al., 2010; Tian et al., 2017).

### Identification of miRNA Target Genes by in Silico and Degradome Approaches

**FIGURE 4:**
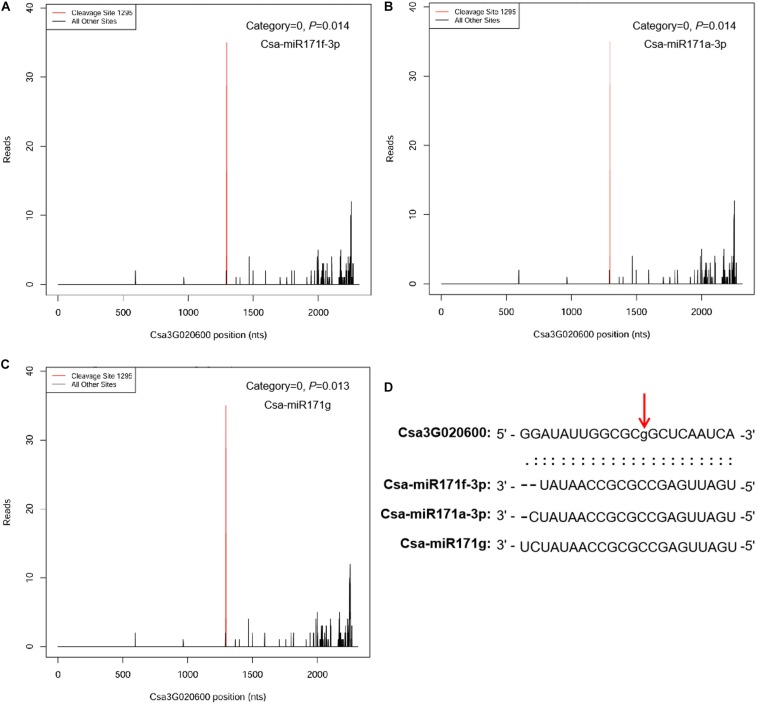
MicroRNAs Csa-miR171f-3p, Csa-miR171a-3p, and Csa-miR171g cotarget *Csa3G020600*. **(A)** Degradome target plot (T-plot) of Csa-miR171f-3p. **(B)** Degradome T-plot of Csa-miR171a-3p. **(C)** Degradome T-plot of Csa-miR171g. **(D)** Alignment of the *Csa3G020600* and mature Csa-miR171f-3p, Csa-miR171a-3p, and Csa-miR171g sequences. The red vertical arrow above the aligned sequences indicates the cleavage site. The *x* axis indicates the positions in the *Csa3G020600* cDNA, and the *y* axis indicates the numbers of degradome sequencing reads.

To identify miRNA targets, we combined target prediction with degradome sequencing. The *in silico* approach predicted 517 target genes for the 32 DEMs in the ID versus NID comparison and 20 target genes for the six DEMs in the IS versus NIS comparison (see Supplementary Table 3 for details). Ten predicted targets, *Csa2G423580*, *Csa5G497010*, *Csa3G144230*, *Csa6G298490*, *Csa1G153530*, *Csa2G215520*, *Csa7G447980*, *Csa6G510860*, *Csa2G076520*, and *Csa3G872160*, were common in the two comparisons. They were targeted by Csa-miR395a-3p and Csa-miR395d-3p in the ID versus NID and IS versus NIS comparisons, respectively. The highest number of targets was identified for Csa-miR172c-3p (139 genes), followed by Csa-miR156g (53 genes) and Csa-miR396d-3p (50 genes). Four degradome cDNA libraries (NID, ID, NIS, IS) were constructed and sequenced to detect miRNA-guided cleavage products. A total of 16,479,774 degradome tags were obtained and mapped to the cucumber genome (9930 V2.0), with approximately 59.7% (over 9.8 million) matching perfectly to the genome. Based on the strength of the degradome signal at the miRNA target sites, a total of 67 target genes corresponding to 101 miRNAs were identified (*p* < 0.05) (Supplementary Table 4). The 67 target genes fell into three categories (0, 1, and 3). Approximately 85% (57 genes) of them were in category 0, which represents the most abundant degradome tags corresponding to the cleavage site and matching cognate transcripts (Zhu et al., 2019). The highest number of targets (60) was identified for IS, and the lowest number was identified for NIS (35). All the DEM–target pairs identified by degradome sequencing were among those predicted by psRNATarget. These two methods also showed that one target gene could be cleaved by multiply miRNAs. For example, *Csa3G020600* (a GRAS TF) was cotargeted by Csa-miR171f-3p, Csa-miR171a-3p, and Csa-miR171g at the same location (Figure 4).

**FIGURE 5:**
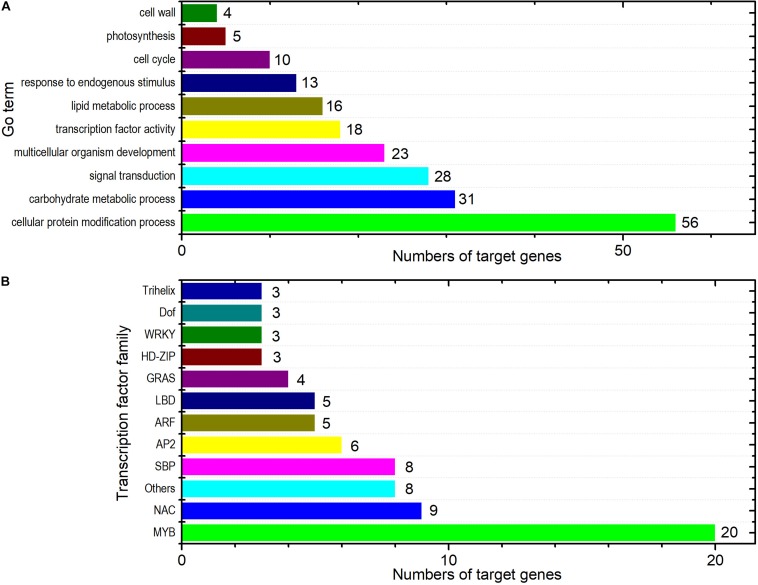
Functional annotation of the predicted target genes of the DEMs. **(A)** Selected GO terms that were significantly enriched among the predicted target genes. **(B)** Graphical representation of the target genes annotated as TFs under their assigned TF families. Other TF families among the target genes included BES1, bHLH, bZIP, C2H2, C3H, CO-like, FAR1, HB-PHD, Nin-like, and TALE. Detailed information about these families is available in the plant TF database V3.0 (http://planttfdb.cbi.pku.edu.cn/).

Gene ontology slim classification analysis was carried out to elucidate the potential functions of the DEM targets in response to PM inoculation. We obtained 86 different GO annotations for the target genes (Supplementary Table 5). The GO terms including carbohydrate metabolic process (GO:0005975, 31 genes), lipid metabolic process (GO:0006629, 16 genes), photosynthesis (GO:0015979, five genes), signal transduction (GO:0007165, 28 genes), and response to endogenous stimulus (GO:0009719, 13 genes) were among others (Figure 5A). It has been suggested that miRNAs may provide genetic switch mechanisms to essentially inactivate the target genes by regulation of TF functioning and TF-mediated events (Chen et al., 2011; Arora et al., 2013). In total, 77 targets belonging to 19 TF families were detected, which accounted for nearly 15% of the target genes of the 32 DEMs in ID versus NID. These TFs included members of the MYB (20 genes), NAC (nine genes), and SBP (eight genes) families (Figure 5B; see Supplementary Table 6 for details).

### Negatively Regulated miRNA Target Genes

Given that miRNAs negatively regulate their targets, we considered that true targets will be downregulated when the miRNA is upregulated under the same PM-inoculation treatment and vice versa (Xu et al., 2019). Thus, the expression of PM-responsive DEMs (from the current sRNA-seq data) and their target genes (from the published RNA-seq study that had the same experimental design, GSE81234) were compared. We identified 92 and three miRNA–mRNA interaction pairs in D8 and SSSL508-28, respectively. A complete list of these DEMs and their negatively regulated targets (*p* < 0.05) with the log2 fold changes and annotations can be found in Supplementary Table 7. The identified miRNA–mRNA pairs accounted for only a small proportion of the predicted targets. The remaining targets either showed no significant change in their expression levels or had similar expression patterns as the corresponding miRNAs.

Among the 92 miRNA–mRNA interaction pairs in D8, two were located on the substituted segment (Chr5:16,676,542–23,484,079 bp; Xu et al., 2017) that includes Csa-miR172c-3p and Csa-miR395a-3p. Eight targets of Csa-miR172c-3p that were negatively regulated upon PM inoculation were classified as TFs, namely, two Dofs (*Csa1G009790* and *Csa1G033250*), one TALE (*Csa6G426360*), one AP2 (*Csa2G279250*), one LBD (*Csa5G219380*), one Trihelix (*Csa2G350410*), one bHLH (*Csa1G612950*), and one CO-like (*Csa7G031530*) (Supplementary Table 7). Among the three miRNA–mRNA interaction pairs in SSSL508-28, three of the DEMs were located on the substituted segment (Csa-miR395d-3p, Csa-miR398d, and Csa-miR398b-3p), and their targets included a polygalacturonase (PG, *Csa7G433170*) and eukaryotic translation initiation factor 2α (eIF2α, *Csa4G011690*). A gene encoding UDP-glycosyltransferase 1 (UTG, *Csa1G526840*) was the target of Csa-miR398b-3p. Only one negatively regulated target of Csa-miR162a, *Csa5G524850* (L-aspartate oxidase, Chr5:18,665,821–18,670,912 bp), was located on the substituted segment.

**FIGURE 6:**
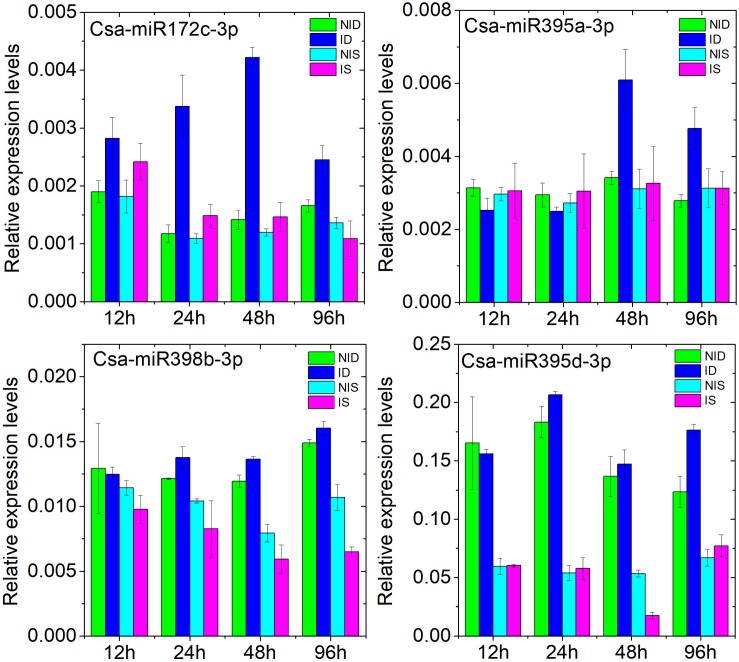
Analysis of Csa-miR172c-3p, Csa-miR395a-3p, Csa-miR395d-3p, and Csa-miR398b-3p at 12, 24, 48, and 96 h after PM inoculation in cucumber SSSL508-28 and D8 lines by stem-loop qRT-PCR. NID, non-inoculated D8 control leaves; ID, PM-inoculated D8 leaves; NIS, non-inoculated SSL508-28 control leaves; IS, PM-inoculated SSL508-28 leaves. Data are the means of three replicates (± SD). The U6 gene was used as an internal control to normalize the expression data.

We further investigated the expression dynamics of Csa-miR172c-3p, Csa-miR395a-3p, Csa-miR395d-3p, and Csa-miR398b-3p at 12, 24, 48, and 96 h after PM inoculation by stem-loop qRT-PCR. The details are presented in Figure 6. Csa-miR172c-3p and Csa-miR395a-3p shared similar expression patterns in response to PM inoculation in D8 as compared with SSL508-28. Csa-miR395d-3p was downregulated in SSL508-28 at 48 h and upregulated in D8 at 96 h after PM inoculation. Csa-miR398b-3p was repressed congruously in PM-inoculated SSL508-28. These results further confirmed that these DEMs may be involved in the cucumber–PM interaction.

## Discussion

The role of miRNAs in the PM response has been reported in wheat (Xin et al., 2010; Wu et al., 2015) and barely (Hunt et al., 2019), but the relationship between miRNAs and cucumber PM resistance is still not clear. The scanning electron microscopy results showed a higher density of PM hyphae on the leaf surface of D8 than on SSSL508-28 48 h after inoculation (Figure 1), which reflected their different responses to PM. To better understand the molecular mechanisms behind PM resistance in cucumber, we sequenced sRNA and degradome libraries constructed from the PM-infected leaves of D8 and SSSL508-28 at 48 h after treatment and the corresponding non-infected controls. The comparative analysis identified 32 and six PM-responsive DEMs in D8 and SSSL508-28, respectively. More DEMs were identified in the ID versus NID comparison (approximately 5.4-fold) than in the IS versus NIS comparison (Tables 3, 4), which suggested the miRNA expression levels may vary greatly depending on the lines/varieties. All of the 32 DEMs identified in the PM-susceptible D8 were upregulated, whereas most of the DEMs identified in the PM-resistant SSSL508-28 were downregulated. Interestingly, several of these PM-related miRNAs have been reported previously. For instances, Han et al. (2016) found the accumulation of vvi-miR156f in PM-inoculated Chinese wild *Vitis pseudoreticulata* leaves, and Jagadeeswaran et al. (2009) found miR398 expression decreased in *Arabidopsis* leaves infiltrated with a virulent strains of *Pseudomonas syringae* pv. *tomato*, Pst DC3000. In the current study, we found that several miR156 variants (Cs-miR156j, Cs-miR156k, Cs-miR156, and Cs-miR156 g) were upregulated in PM-inoculated cucumber D8 leaves, which is consistent with the previous studies. In addition, Csa-miR398b-3p and Csa-miR398c were downregulated upon PM inoculation only in SSSL508-28. These results suggested some miRNAs may have similar expression patterns in disease resistance among different plant species. However, many of the DEMs identified in our study were not significantly affected or showed opposite trends to those reported by Xin et al. (2010) and Han et al. (2016), including members of the miR398 (Csa-miR398a-3p and Csa-miR398d) and miR858 (Csa-miR858a and Csa-miR858b) families. These discrepancies suggest that some miRNAs may be species-specific in response to PM inoculation or are expressed at specific developmental stages or in specific tissues. Thus, the expression patterns of these miRNAs need to be studied individually.

MicroRNAs respond to environmental stresses by degrading or inhibiting the translation of their target genes. We obtained more than 500 target genes with diverse functions for the identified DEMs using computational and degradome approaches. The enrichment of GO terms such as regulation of RNA biosynthetic process (GO:2001141) and defense response (GO:0006952) highlighted the importance of these targets in the PM response (Figure 5A). Generally, the accumulation of miRNAs silences their target genes and vice versa (Xu et al., 2019). To reduce the number of false positives and obtain a more mechanistic insight about the regulatory activity of the cucumber DEMs, we focused on those that targeted negatively expressed genes with transcriptomic evidence. The DEMs and/or target genes within the substituted segment of SSL508-28 might be used to prioritize genetic factors because they directly affect phenotypic variation. Csa-miR172c-3p, which is located on the substituted segment, is of particular interest because it accumulated in PM-inoculated D8 leaves and targeted TFs. It is conceivable that miRNAs that target TFs will have an extensive influence on gene expression. We found that Csa-miR172c-3p regulated TFs such as AP2 (*Csa2G279250*), bHLH (*Csa1G612950*), and Dofs (*Csa1G009790* and *Csa1G033250*), which have been shown to play important roles in disease responses in plants (Samad et al., 2017; Kessens et al., 2018). Further, expression profiling studies have shown that some of these TFs positively regulate plant immune responses. For example, in barley, several AP2 TFs were upregulated at 12 h after PM inoculation (Molitor et al., 2011), and in the pumpkin resistant line “112-2”, *bHLH61* (c71304_g1) was found to be induced at 3, 6, 24, and 48 h after PM infection (Guo et al., 2018). Moreover, seven Dofs (*CsDof27*, *CsDof29*, *CsDof03*, *CsDof18*, *CsDof28*, *CsDof35*, and *CsDof36*) from cucumber and one from grape (*VvDOF3*) were found to be induced by inoculation with the downy mildew pathogen and PM, respectively (Wen et al., 2016; Yu et al., 2019). Thus, we hypothesized that, in D8 leaves, the specific downregulation of the TFs targeted by Csa-miR172c-3p may at least partially account for the low PM resistance of the cucumber D8 line. Besides the TFs, Csa-miR172c-3p also targets *Csa6G062300*, which encodes a type 2c protein phosphatase (PP2C). Protein phosphatases are the obligate partners of protein kinases in the cellular control circuitry (DeLong, 2006). Biological functions have been assigned to several PP2Cs, including organ development (Lita et al., 2003), MAPK signaling (Schweighofer et al., 2004), ABA perception (Park et al., 2009), and disease resistance (Widjaja et al., 2010). Hu et al. (2009) found that ectopic expression of a rice PP2C gene *OsBIPP2C2a* in tobacco plants resulted in enhanced disease resistance to *Phytophthora parasitica* var. *nicotianae* and mosaic virus and constitutive expression of defense-related genes. The PP2C identified in this work, *Csa6G062300*, was downregulated in D8 after PM inoculation, implying that phosphatase levels regulated by Csa-miR172c may be important for cucumber PM resistance. However, little is known about the two negatively regulated targets of Csa-miR395a-3p, *Csa3G020600* (encoding a zinc finger protein), and *Csa4G664250* (encoding a coiled-coil domain-containing protein).

Despite the limited number of miRNA–mRNA interaction pairs that were identified in SSL508-28, two of the DEMs (Csa-miR395d-3p and Csa-miR398b-3p) were located on the substituted segment, and many predicted targets have been reported previously to be involved in disease resistance. An inverse expression pattern was found for Csa-miR395d-3p and its target gene *Csa4G011690*, which encodes the eukaryotic translation initiation factor 2α (eIF2α). eIF2α is required in the initiation of the translation process because it regulates the rate of global protein synthesis (Chambers et al., 2015; Sesma et al., 2017). Enhanced global protein synthesis is cytoprotective in the early response to many biotic stresses. The downregulation of Csa-miR395d-3 and upregulation of *eIF2*α in SSSL508-28 suggested that the resistant genotype could activate protein synthesis of, for example, receptor-like kinases and receptor-like proteins, at an early stage of infection to restrict the growth of PM pathogens as was reported previously (Tang et al., 2017). Csa-miR398b-3p was found to negatively regulate its target *Csa1G526840*, which encodes UGT, in response to PM inoculation. This role of Csa-miR398b has not been reported previously. UGTs encode cytosol-localized enzymes that catalyze the conjugation of a range of diverse small lipophilic compounds with sugars to generate water-soluble glycosides (Ahn et al., 2012). The importance of UGTs in plant defense responses against infections has been well characterized. For example, overexpression of *UGT74F2* and *UGT76B1*, or knockout of *ugt73b3* and *ugt73b5*, significantly altered plant resistance to pathogen infection (Langlois-Meurinne et al., 2005; Song et al., 2008; von Saint Paul et al., 2011). Xing et al. (2018) found that wheat overexpressing *UGT3* exhibited significantly enhanced resistance to *Fusarium* head blight and increased the endogenous salicylic acid and jasmonic acid content in the grains compared with in the untransformed control. Therefore, the enhanced expression of the UGT (*Csa1G526840*) genes in the resistant SSSL508-28 genotype compared with their expression in the susceptible D8 genotype might be associated with PM resistance.

One of the negatively regulated targets of Csa-miR162a, L-aspartate oxidase (LASPO, Csa5G524850), also was located on the substituted segment and was specifically downregulated in PM-inoculated D8 leaves (Supplementary Table 7). Although LASPO is the first enzyme in the *de novo* biosynthesis of NAD^+^ pathway in plants, little is known about its biochemical properties in plants. Pétriacq et al. (2012) reported the upregulation of *AtLASPO* (*At5g14760*) transcripts in *Arabidopsis* leaves infected with the hemibiotrophic bacterial plant pathogen *P. syringae*. Macho et al. (2012) observed that the impaired stomatal immunity against *P. syringa* was caused by mutations in the gene encoding *AtLASPO*. Hao et al. (2018) found that *AtLASPO* expression was clearly correlated with LASPO activity and NAD^+^ levels. These results suggested a pivotal role for LASPO in NAD^+^ production upon pathogen infection. We speculated that downregulation of cucumber *LASPO* by Csa-miR162a in D8 may be another reason for its sensitivity to PM.

## Conclusion

Key miRNA–target regulatory pairs were identified in cucumber in response to PM infection by sRNA transcriptomics and degradome sequencing. A total of 32 and six DEMs were identified in the ID versus NID and IS versus NIS comparisons, respectively. The integrated analysis of the expression data identified a total of 92 and three miRNA–mRNA interaction pairs in D8 and SSSL508-28, respectively, which showed inverse expression patterns. The comparative study highlighted an extensive genotype-specific response to PM infection that was different from the common responses in the resistant SSSL508-28 and susceptible D8 genotypes. The miRNAs Csa-miR172c-3p, Csa-miR395a-3p, Csa-miR395d-3p, and Csa-miR398b-3p and the target genes *AP2*, *bHLH*, *Dof*, *UGT*, and *LASPO* were found to play critical roles in the PM-inoculated cucumber leaves. Further studies of these miRNAs and target genes will further expand our understanding of the molecular mechanisms underlying PM resistance in cucumber.

## Data Availability Statement

The raw small RNA sequencing reads were deposited into NCBI Sequence Read Archive (SRA) under the accession number PRJNA563503. The raw degradome RNA sequencing reads were deposited into NCBI SRA under the accession number PRJNA574048.

## Author Contributions

XC conceived and designed the study. XX analyzed the sequencing data and wrote the manuscript. CZ collected the samples and performed the qRT-PCR study. MT, YS, XX, XQ, and QX helped analyze the data and revise the manuscript. All authors reviewed and approved this submission.

## Conflict of Interest

The authors declare that the research was conducted in the absence of any commercial or financial relationships that could be construed as a potential conflict of interest.

## Footnotes

1 http://cucurbitgenomics.org/

2 http://rfam.xfam.org/

3 https://www.arb-silva.de/

4 http://gtrnadb.ucsc.edu/

5 https://www.girinst.org/

6 http://www.mirbase.org/

7 http://plantgrn.noble.org/psRNATarget

8 http://cucurbitgenomics.org/funcat
